# The Impact of Month on Joint Arthroplasty In-Hospital Outcomes: The December Effect

**DOI:** 10.1016/j.artd.2021.12.006

**Published:** 2022-06-01

**Authors:** Leo Zalikha, Kassem-Ali J. Abbas, Patrick Karabon, Inaya Hajj Hussein, Mouhanad M. El-Othmani

**Affiliations:** a Department of Orthopaedic Surgery and Sports Medicine, Detroit Medical Center, Detroit, MI, USA; b College of Medicine and Life Sciences, University of Toledo, Toledo, OH, USA; c Department of Foundational Medical Studies, Oakland University William Beaumont School of Medicine, Auburn Hills, MI, USA

## Abstract

**Background:**

The purpose of this study was to assess the impact of month of the year on postsurgical outcomes after primary total hip arthroplasty (THA) and total knee arthroplasty (TKA) and to specifically analyze for a December effect.

**Material and methods:**

The National Inpatient Sample was used to identify all patients older than 40 years undergoing primary TKA and THA between 2006 and 2015. Patients were stratified based on the month of the year of surgery. In-hospital complication, disposition, and economic outcomes were comparatively analyzed.

**Results:**

There were statistically significant differences in outcomes based on month of the year. When comparing December to the other months, both TKA and THA patients had significantly lower rates of any complication, postoperative anemia, and genitourinary complications, while there were significantly higher rates of home than rehab discharge and shorter average length of stay in December. THA patients additionally had significantly lower rates of cardiac and respiratory complications during December.

**Conclusion:**

Postoperative outcomes are significantly associated with the month in which arthroplasty is performed. This study provides evidence of a positive “December effect” of improved in-hospital complications and economic outcomes for surgeries performed in December. Future research should direct attention to the impact that social factors may have on outcomes after elective surgical procedures and how these factors may be translated to other months.

## Introduction

Primary total hip arthroplasty (THA) and total knee arthroplasty (TKA) are among the most common surgical procedures in the United States. [1] Although these procedures are widely regarded as safe and effective, their postoperative complications are associated with significant patient morbidity and financial burden to the health-care system [2]. While substantial effort has been made on assessing the impact of medical comorbidities and modifiable risk factors on outcomes after these procedures, far less attention has been placed on the potential impact of situational and occasional factors, such as month of the year, on outcomes after total joint arthroplasty (TJA).

The potential impact of season and month of the year on postoperative outcomes has been examined in the general surgical and orthopedic literature [[3], [4], [5], [6]]. Within the orthopedic literature, most studies analyzing the impact of month have focused on the July effect, which is a controversial concept representing a theorized disruption in the health-care system because of the influx and advancement of inexperienced trainees to positions with heightened clinical responsibilities. In the TJA literature, most large-registry studies have been unable to demonstrate any evidence of a July effect [[7], [8], [9]]. In contrast, some studies have demonstrated seasonal variations in certain postoperative outcomes, although their results are variable [[4], [5], [6]]. However, because such seasonal studies typically analyze data pooled by yearly quarters or seasons, the assessment of the unique impact of specific months remains lacking. The understanding of monthly variation in outcomes is important since the December holiday season has been identified as a risk factor in the medical literature for unfavorable health outcomes and worsened patient morbidity and mortality [[10], [11], [12]]. Because of anecdotal and such clinical data suggesting that December and its associated holiday season may impact medical and surgical outcomes, analyzing the impact of the month of December specifically on in-hospital outcomes after TJA is needed to identify potential unique risk factors, which may uncover addressable opportunities for better risk stratification and improved postsurgical outcomes.

In that context, the purpose of this study was to (1) provide a comprehensive description of in-hospital complications, resources utilization, and discharge dispositions based on the month of the year in TJA patients and (2) evaluate for a potential December effect in these in-hospital outcomes.

## Material and methods

Discharge data from 2006 to the third quarter of 2015 from the National Inpatient Sample (NIS) were collected and retrospectively analyzed. The NIS, a part of the Healthcare Cost and Utilization Project, is the largest all-payer inpatient hospital database in the United States. It uses a 20% stratified sample of discharges from U.S. hospitals and is weighted to provide precise estimates at the national level. *The International Classification of Disease, Ninth Revision, Clinical Modification* was used for procedure and diagnosis codes during the period of this study. Institutional review board exemption was approved for this study.

Patients older than 40 years who underwent a primary TKA (*International Classification of Disease* code 81.54) or primary THA (81.51) were included in this study. Patients younger than 40 years and those undergoing revision procedures were excluded. TJA patients were considered to be those who underwent either a primary TKA or THA. In accordance with recommendations from the Agency for Healthcare Research and Quality, discharge weights, clusters, and strata were all accounted for, and a proper domain and subanalysis was performed [13,14]. After identification of the TJA cohorts, patients were stratified into groups based on admission month using the 12 months of the year. Because data from both before and after the 2012 NIS redesign were used, NIS trend weights were used for this analysis [15].

Analyzed outcomes included cardiac, peripheral vascular disease, respiratory, gastrointestinal (GI), genitourinary, hematoma/seroma, wound dehiscence, postoperative infection, deep vein thrombosis, pulmonary embolism, and postoperative anemia complications. The variable “any complication” was used as a composite measure referring to any of these complications. Patient demographics, length of stay (LOS), disposition, and charges also were comparatively analyzed. The data were analyzed initially comparing admissions during every month of the year. After surgeries in December were observed to have the lowest overall complication rate, further subanalysis was performed by comparing outcomes during the month of December vs the rest of the months. Complications and discharge disposition were analyzed using complex samples logistic regression while economic outcomes were analyzed using complex samples linear regression. All models were adjusted for Elixhauser comorbidities, which were identified as confounding factors. Adjusted, or marginal, proportions and averages were generated for categorical and continuous outcomes, respectively, for each month along with 95% confidence intervals. Statistical significance was defined at *P* value < .05. All statistical analyses were performed using SAS 9.4 (SAS Institute Inc., Cary, NC) and Stata 13 (StataCorp, LP, College Station, TX).

## Results

### Nationwide TJA rates and patient demographics

A total of 5,623,908 TKAs and 2,707,182 THAs were performed during the study period. The most common month of admission was June (9.00%) while the least common was December (7.21%). A breakdown of patient demographics and hospital descriptive data for all patients included in this study is provided in Table 1.

**Table 1:** Patient characteristics and hospital descriptive data for all TJA patients during the entire study period.

Variable	Discharges (n = 8,330,035)
Age of patient (mean y, standard error)	66.11 (0.03)
Biological sex of patient	
Male	3,272,842 (39.29%)
Female	5,057,193 (60.71%)
Expected primary payor	
Medicare	4,516,470 (54.22%)
Medicaid	264,463 (3.18%)
Private insurance	3,231,675 (38.80%)
Self-pay	44,441 (0.53%)
No charge	7380 (0.09%)
Other insurance	265,606 (3.19%)
Race of patient	
Non-Hispanic white	5,996,065 (71.98%)
Non-Hispanic black	524,848 (6.30%)
Hispanic	326,014 (3.91%)
Other race	1,483,108 (17.80%)
Total hip arthroplasty	2,707,182 (32.50%)
Total knee arthroplasty	5,623,908 (67.51%)
Admission month	
January	764,033 (9.17%)
February	682,481 (8.19%)
March	713,667 (8.57%)
April	702,335 (8.43%)
May	678,983 (8.15%)
June	749,805 (9.00%)
July	689,503 (8.28%)
August	689,237 (8.27%)
September	691,809 (8.31%)
October	715,481 (8.59%)
November	651,107 (7.83%)
December	600,594 (7.21%)
Bedsize of hospital	
Small	1,687,736 (20.26%)
Medium	2,210,452 (26.54%)
Large	4,402,229 (52.85%)
Unknown	29,618 (0.36%)
Location/teaching status of hospital	
Rural	954,870 (11.46%)
Urban nonteaching	3,465,596 (41.60%)
Urban teaching	3,879,950 (46.58%)
Unknown	29,618 (0.36%)
Region of hospital	
Northeast	1,552,229 (18.63%)
Midwest	2,363,455 (28.37%)
South	2,689,488 (32.29%)
West	1,724,863 (20.71%)

### Comprehensive TJA monthly in-hospital outcomes analysis

In-hospital postoperative complications and discharge disposition for the TJA patient cohort significantly differed based on month of the year. For all TJA patients, there were statistically significant differences in the rates of any (*P* < .001), respiratory (*P* < .001), GI (*P* < .001), wound dehiscence (*P* = .02), deep vein thrombosis (*P* = .03), pulmonary embolism (*P* = .01), and postoperative anemia (*P* < .001) complications based on the month of the year. There were no statistically significant differences in the rates of cardiac, peripheral vascular disease, genitourinary, hematoma/seroma, and postoperative infection complications within the TJA cohort. There were statistically significant differences in the rates of home vs rehabilitation (rehab) discharge (*P* < .001), LOS (*P* < .001), and total charges (*P* < .001) based on the month of the year. Among all months, the lowest rate of any complication was observed during December (*P* < .001). A description of TJA in-hospital complications and economic and disposition outcomes are given in Tables 2 and 3, respectively.

**Table 2:** Adjusted rates of all in-hospital complications for TJA patients, stratified by month of the year.

Month	Any *P* < .001	Cardiac *P* = .67	PVD *P* = .35	Respiratory *P* < .001	GI *P* < .001	GU *P* = .37	Hematoma/Seroma *P* = .051	Wound dehisce *P* = .02	Infection *P* = .12	DVT *P* = .03	PE *P* = .01	Anemia *P* < .001
January	23.72% (23.07%, 24.38%)	0.63% (0.58%, 0.67%)	0.10% (0.08%, 0.12%)	0.19% (0.17%, 0.22%)	0.27% (0.24%, 0.30%)	0.52% (0.48%, 0.56%)	0.64% (0.59%, 0.70%)	0.10% (0.08%, 0.12%)	0.13% (0.11%, 0.15%)	0.34% (0.31%, 0.38%)	0.35% (0.33%, 0.37%)	21.88% (21.21%, 22.55%)
February	23.82% (23.15%, 24.49%)	0.65% (0.60%, 0.69%)	0.12% (0.10%, 0.14%)	0.19% (0.16%, 0.21%)	0.25% (0.22%, 0.28%)	0.51% (0.47%, 0.55%)	0.63% (0.57%, 0.68%)	0.09% (0.07%, 0.10%)	0.14% (0.12%, 0.16%)	0.37% (0.33%, 0.41%)	0.36% (0.34%, 0.39%)	22.01% (21.33%, 22.69%)
March	24.07% (23.39%, 24.76%)	0.65% (0.61%, 0.70%)	0.11% (0.09%, 0.12%)	0.19% (0.16%, 0.22%)	0.29% (0.26%, 0.32%)	0.53% (0.48%, 0.57%)	0.62% (0.56%, 0.68%)	0.08% (0.07%, 0.10%)	0.14% (0.11%, 0.16%)	0.31% (0.28%, 0.35%)	0.34% (0.32%, 0.37%)	22.27% (21.58%, 22.97%)
April	23.92% (23.25%, 24.58%)	0.63% (0.58%, 0.67%)	0.11% (0.09%, 0.13%)	0.18% (0.15%, 0.20%)	0.27% (0.24%, 0.30%)	0.48% (0.44%, 0.53%)	0.65% (0.59%, 0.71%)	0.09% (0.08%, 0.11%)	0.14% (0.12%, 0.16%)	0.32% (0.29%, 0.35%)	0.30% (0.28%, 0.33%)	22.18% (21.50%, 22.86%)
May	24.21% (23.51%, 24.91%)	0.65% (0.60%, 0.70%)	0.11% (0.09%, 0.13%)	0.20% (0.16%, 0.23%)	0.27% (0.24%, 0.30%)	0.52% (0.48%, 0.57%)	0.70% (0.64%, 0.76%)	0.08% (0.07%, 0.10%)	0.13% (0.11%, 0.14%)	0.31% (0.27%, 0.34%)	0.33% (0.31%, 0.36%)	22.42% (21.71%, 23.13%)
June	24.01% (23.33%, 24.69%)	0.63% (0.59%, 0.68%)	0.10% (0.09%, 0.12%)	0.17% (0.14%, 0.20%)	0.25% (0.23%, 0.28%)	0.52% (0.48%, 0.56%)	0.63% (0.57%, 0.69%)	0.06% (0.05%, 0.08%)	0.13% (0.11%, 0.15%)	0.33% (0.29%, 0.36%)	0.35% (0.33%, 0.37%)	22.27% (21.59%, 22.96%)
July	23.85% (23.17%, 24.54%)	0.64% (0.59%, 0.69%)	0.10% (0.08%, 0.12%)	0.19% (0.16%, 0.21%)	0.22% (0.19%, 0.25%)	0.48% (0.44%, 0.52%)	0.64% (0.58%, 0.69%)	0.09% (0.07%, 0.10%)	0.11% (0.09%, 0.13%)	0.30% (0.26%, 0.33%)	0.33% (0.31%, 0.36%)	22.15% (21.46%, 22.85%)
August	24.06% (23.36%, 24.77%)	0.62% (0.58%, 0.67%)	0.09% (0.07%, 0.10%)	0.17% (0.15%, 0.20%)	0.28% (0.24%, 0.31%)	0.51% (0.46%, 0.56%)	0.60% (0.55%, 0.65%)	0.10% (0.08%, 0.11%)	0.10% (0.08%, 0.12%)	0.30% (0.27%, 0.34%)	0.33% (0.31%, 0.35%)	22.34% (21.63%, 23.05%)
September	24.03% (23.34%, 24.73%)	0.64% (0.59%, 0.68%)	0.10% (0.08%, 0.12%)	0.17% (0.14%, 0.21%)	0.25% (0.22%, 0.28%)	0.47% (0.43%, 0.52%)	0.57% (0.52%, 0.62%)	0.07% (0.06%, 0.09%)	0.12% (0.10%, 0.14%)	0.33% (0.30%, 0.36%)	0.31% (0.29%, 0.34%)	22.36% (21.65%, 23.07%)
October	24.25% (23.49%, 25.01%)	0.64% (0.60%, 0.69%)	0.09% (0.08%, 0.11%)	0.14% (0.12%, 0.16%)	0.24% (0.21%, 0.26%)	0.53% (0.49%, 0.57%)	0.65% (0.60%, 0.70%)	0.09% (0.07%, 0.10%)	0.12% (0.10%, 0.14%)	0.31% (0.28%, 0.34%)	0.33% (0.31%, 0.35%)	22.55% (21.78%, 23.33%)
November	23.92% (23.15%, 24.70%)	0.61% (0.56%, 0.66%)	0.11% (0.09%, 0.12%)	0.13% (0.11%, 0.15%)	0.22% (0.20%, 0.25%)	0.52% (0.48%, 0.57%)	0.60% (0.55%, 0.66%)	0.10% (0.08%, 0.12%)	0.13% (0.11%, 0.15%)	0.31% (0.28%, 0.35%)	0.36% (0.33%, 0.39%)	22.22% (21.44%, 23.01%)
December	23.32% (22.56%, 24.08%)	0.58% (0.54%, 0.63%)	0.10% (0.08%, 0.11%)	0.12% (0.10%, 0.14%)	0.20% (0.17%, 0.22%)	0.50% (0.45%, 0.54%)	0.61% (0.55%, 0.67%)	0.09% (0.07%, 0.10%)	0.13% (0.11%, 0.16%)	0.31% (0.28%, 0.35%)	0.33% (0.31%, 0.36%)	21.64% (20.87%, 22.41%)

DVT, deep vein thrombosis; PE, pulmonary embolism; PVD, peripheral vascular disease.

*P* value represents statistical significance of difference between monthly values. Confidence intervals 95% are given in parentheses.

**Table 3:** Adjusted rates of in-hospital length of stay, disposition, and economic outcomes for all TJA patients, stratified by month of the year.

Month	Home discharge, *P* < .001	Rehab discharge, *P* < .001	Average LOS (d), *P* < .001	Total charges, *P* < .001
January	66.36% (65.68%, 67.04%)	32.66% (31.95%, 33.38%)	3.29 (3.26, 3.32)	$50,144 ($49,308, $50,981)
February	66.63% (65.96%, 67.29%)	32.42% (31.71%, 33.13%)	3.26 (3.24, 3.28)	$50,514 ($49,684, $51,343)
March	66.38% (65.67%, 67.09%)	32.60% (31.84%, 33.36%)	3.24 (3.21, 3.26)	$50,343 ($49,478, $51,208)
April	65.70% (65.02%, 66.38%)	33.39% (32.65%, 34.12%)	3.21 (3.19, 3.23)	$50,484 ($49,637, $51,331)
May	65.33% (64.62%, 66.04%)	33.60% (32.83%, 34.36%)	3.22 (3.20, 3.25)	$50,440 ($49,590, $51,290)
June	66.38% (65.68%, 67.08%)	32.60% (31.84%, 33.36%)	3.20 (3.18, 3.22)	$50,537 ($49,682, $51,391)
July	66.31% (65.63%, 66.99%)	32.75% (32.02%, 33.48%)	3.20 (3.18, 3.22)	$51,400 ($50,546, $52,254)
August	65.89% (65.19%, 66.58%)	33.15% (32.42%, 33.89%)	3.20 (3.18, 3.23)	$51,258 ($50,384, $52,133)
September	65.76% (65.06%, 66.46%)	33.28% (32.53%, 34.04%)	3.18 (3.16, 3.21)	$50,924 ($50,046, $51,801)
October	65.65% (64.90%, 66.40%)	33.35% (32.54%, 34.15%)	3.25 (3.23, 3.27)	$50,289 ($49,356, $51,221)
November	68.41% (67.68%, 69.13%)	30.67% (29.89%, 31.45%)	3.23 (3.20, 3.26)	$50,340 ($49,397, $51,284)
December	72.10% (71.44%, 72.75%)	27.03% (26.34%, 27.73%)	3.16 (3.14, 3.19)	$50,716 ($49,797, $51,634)

*P* value represents statistical significance of difference between monthly values. Confidence intervals 95% are given in parentheses.

### December vs other months in-hospital outcomes analysis

#### TKA patients—December vs other months

In December, TKA patients had significantly lower rates of any (21.70% vs 22.34%, *P* = .001), cardiac (0.54% vs 0.62%, *P* = .006), respiratory (0.11% vs 0.18%, *P* < .001), GI (0.17% vs 0.23%, *P* < .001), and postoperative anemia (19.94% vs 20.51%, *P* = .004) complications. There were no significant differences in the other individual complication outcomes. Table 4 provides a complete description of outcomes for TKA and THA patients, stratified by December versus the rest of the year.

**Table 4:** Adjusted TJA (THA + TKA) outcomes, stratified by December vs rest of the year.

	December	Rest of Year	*P*-Value
Any complications	23.32% (22.56%, 24.08%)	23.99% (23.34%, 24.64%)	.0002
Cardiac complication	0.58% (0.54%, 0.63%)	0.64% (0.61%, 0.66%)	.0302
Peripheral vascular disease (PVD) complication	0.10% (0.08%, 0.11%)	0.10% (0.09%, 0.11%)	.4172
Respiratory complication	0.12% (0.10%, 0.14%)	0.18% (0.16%, 0.19%)	<.0001
Gastrointestinal (GI) complication	0.20% (0.17%, 0.23%)	0.26% (0.24%, 0.27%)	.0001
Genitourinary (GU) complication	0.50% (0.45%, 0.54%)	0.51% (0.48%, 0.53%)	.5699
Hematoma/Seroma	0.61% (0.55%, 0.67%)	0.63% (0.60%, 0.67%)	.4786
Wound dehiscence	0.09% (0.07%, 0.10%)	0.09% (0.08%, 0.09%)	.9483
Postoperative infection	0.13% (0.11%, 0.16%)	0.12% (0.12%, 0.13%)	.3598
Deep vein thrombosis (DVT)	0.31% (0.28%, 0.35%)	0.32% (0.30%, 0.34%)	.6677
Pulmonary embolism (PE)	0.33% (0.31%, 0.36%)	0.34% (0.33%, 0.35%)	.8004
Postoperative anemia	21.64% (20.87%, 22.41%)	22.24% (21.58%, 22.90%)	.0008
Home discharge	72.10% (71.44%, 72.75%)	66.24% (65.59%, 66.90%)	<.0001
Rehab discharge	27.03% (26.34%, 27.73%)	32.78% (32.07%, 33.49%)	<.0001
Died during hospitalization	0.09% (0.07%, 0.11%)	0.09% (0.09%, 0.10%)	.8726
Length of stay (LOS)	3.16 (3.14, 3.19)	3.23 (3.21, 3.25)	.0001
Total charges ($)	$50,716 ($49,798, $51,635)	$50,601 ($49,749, $51,453)	.3793

**Figure 1:**
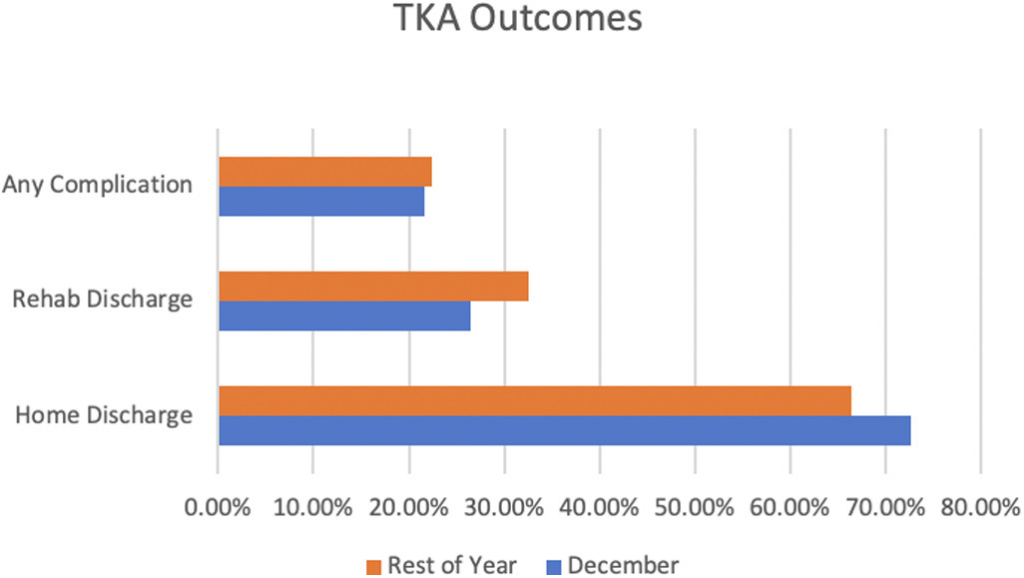
Selected in-hospital outcomes for TKA patients stratified by December vs the rest of the year. All differences are significant.

TKA patients had significantly higher rates of home discharge (72.71% vs 66.44%, *P* < .001), lower rates of rehab discharge (26.46% vs 32.62%, *P* < .001), and shorter LOS (3.13 vs 3.20 days, *P* < .001) in December than in other months. There was no significant difference in total charges in December vs rest of the year ($49,558 vs $49,468, *P* = .53) for TKA patients. Figure 1 provides a snapshot of selected outcomes data for TKA patients in December compared with the rest of the year.

#### THA patients—December vs other months

In December, THA patients had significantly lower rates of any (26.61% vs 27.38%, *P* = .003), GI (0.25% vs 0.31%, *P* = .03), and postoperative anemia (25.11% vs 25.82%, *P* = .006) complications than the other months. There were no significant differences in the other individual complication outcomes.

**Figure 2:**
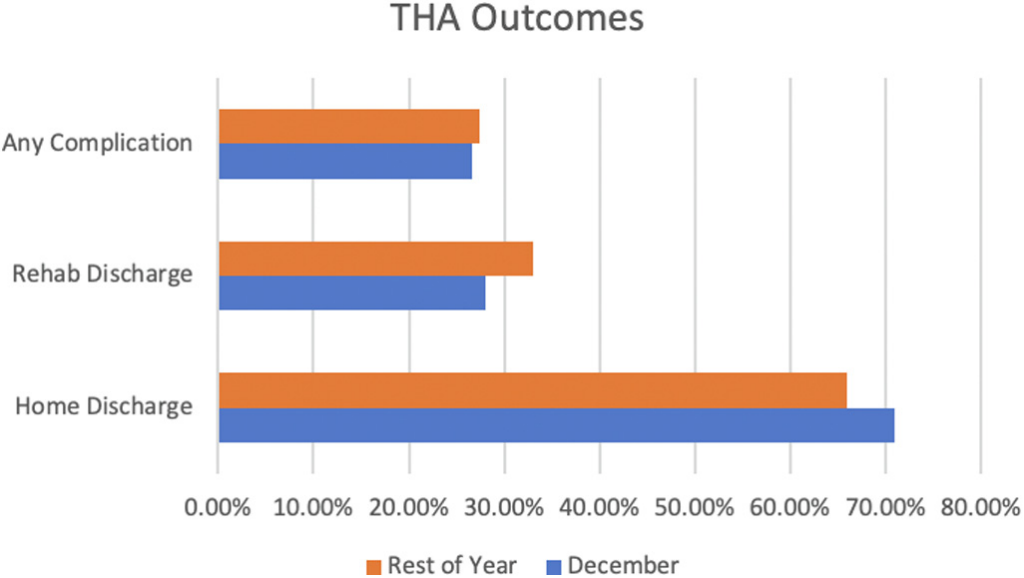
Selected in-hospital outcomes for THA patients stratified by December vs the rest of the year. All differences are significant.

With respect to economic and disposition outcomes, THA recipients had significantly higher rates of home discharge (70.97% vs 65.93%, *P* < .001), lower rates of rehab discharge (28.10% vs 33.02%, *P* < .001), and shorter LOS (3.23 vs 3.27 days, *P* = .001) in December. There was no significant difference in total charges ($53,079 vs $52,950, *P* = .51) in December vs those in other months for THA patients. Figure 2 provides a snapshot of selected outcomes data for THA patients in December compared with the rest of the year.

## Discussion

While previous studies have investigated the influence of season on postoperative outcomes after TJA, their results and conclusions have been variable [[4], [5], [6], [7], [8]]. A large database study of THA patients found no difference in 19 of 20 complication rates when compared by yearly quarter [5]. In contrast, a small retrospective study of 725 TKA patients demonstrated a seasonal effect of inpatient complication rates, with the highest rate in their May-August cohort [4]. Because such seasonal studies are typically analyzed by yearly seasons or quarters, they may be unable to assess the impact of the unique circumstances of specific months. As such, the goal of this study was to investigate the relationship of month of the year on in-hospital postoperative outcomes after TKA and THA and to provide a comprehensive empirical monthly analysis of in-hospital postoperative outcomes with a large national sample of patients. After December was found to have the lowest overall complication rate, a particular focus was directed to analyze outcomes in December compared with the rest of the months. This analysis of December was noteworthy given the disruptions in health outcomes in December that have been previously described in the general medical literature [[10], [11], [12]].

Our analysis revealed marked and consistent evidence of lower immediate in-hospital complication rates, shorter LOS, and increased rates of home compared with rehab discharge during the month of December when compared with rest of the year for both TKA and THA cohorts. Across all months, the lowest rate of any complication, the shortest LOS, and the highest rates of home discharge were observed during December, and these observations were statistically significant. Cardiac, respiratory, GI, and postoperative anemia complications were also lowest during December compared with those in the rest of the year.

There are several plausible social and societal explanations for this December effect, which is opposite to the unfavorable health outcomes during the December holidays that have been documented in the general medical literature [[10], [11], [12]]. Although the evidence base is evolving, patient and family engagement is a marker of high-quality health care and is associated with improved health outcomes and lower use of health services [16]. Notably, the beneficial impact of family support on postoperative outcomes has been previously demonstrated, with higher scores on a family cohesion scale being associated with better postsurgical recovery after open cholecystectomy [17]. In addition, a recent systematic review noted an association of social support and improved patient-reported outcomes after joint replacement [18]. In the United States, December holds major holidays, during which families gather and time off is provided from work and school [19]. It is likely that during this time, patients are afforded increased levels of social support and family engagement perioperatively. This enhanced engagement is likely to manifest both during preoperative clinical care and surgical optimization and during the immediate postoperative inpatient period. Practically, patients may also have increased assistance at home during this time, allowing for increased rates of home discharge compared with rehab discharge. In addition, patients may be incentivized in December to return home quicker to spend the holiday with their family members. Given that nearly 40% of the total cost after an episode of care after TJA occurs after hospital discharge, all these possibilities present a marked potential for cost-saving after TJA [20,21]. The decreased complications rates, decreased costs, and increased rates of home discharge warrant further investigation to develop a deeper understanding of the specific circumstances experienced during December that lead to improved in-hospital outcomes.

Previous studies examining time or month of the year on outcomes after TJA have typically analyzed outcomes by season or focused on the July effect. The July effect is the theorized disruption of the health-care system with an associated increase in medical errors, adverse events, or complications due to an influx of clinically inexperienced residents, fellows, nurses, and physician assistants during the summer months [22]. The July effect on TJA outcomes is questionable, as prior database studies reported no evidence of its existence [7,9]. A similar “August effect” has been hypothesized, related to when adult reconstruction fellows begin their fellowship training; however, although one study demonstrated an August effect on clinical and pain outcomes but not TKA survivorship or complications, most studies have found little evidence of an August effect in arthroplasty [23,24]. Overall, evidence for a July or August effect in TJA is minimal and remains controversial. Similarly, when looking at overall complication rates, this study found that July ranked 4th and August ranked 9th out of the 12 months in terms of the lowest overall complication rate. This finding seems to support previous literature, which has shown little empirical evidence of a detrimental July or August effect on outcomes after TJA.

This study has limitations, several of which are inherent to large database studies. Although they provide an immense volume of data, national databases are prone to errors and incompleteness [25]. Despite this, Bozic et al. found that comorbidity and complication data from administrative records are accurate [26]. In addition, many studies have used and validated such registries for reporting in-hospital outcomes [27,28]. In addition, although the improved immediate in-hospital outcomes this study documented during December are promising and valuable, this study was not designed to report on long-term outcomes. Future studies are needed to determine if the improved in-hospital outcomes observed during December are also sustained as improved long-term outcomes.

Despite these limitations, there were several important strengths to this study. This study provides a comprehensive, epidemiological observational snapshot of multiple in-hospital outcomes after TJA by every month of the year over an extended duration of time. This study additionally demonstrated a novel positive December effect in a wide range of in-hospital outcomes, including complication rates, LOS, and discharge disposition, consistently for the entire TJA group as well as for the TKA and THA cohorts separately. The applied statistical method used also strengthened the findings of this study, as it allowed for controlling for potentially confounding comorbidity variables. These findings might indicate a potentially critical role for social factors in determining outcome measures and quality metrics, such as LOS and discharge disposition, and warrant further investigation and consideration for inclusion as part of risk-stratification tools.

## Conclusions

This study provided evidence of a beneficial December effect of decreased in-hospital postoperative complications, decreased LOS, and increased rates of home compared with rehab discharge. As health-care systems shift toward value-based delivery models, researchers and clinicians continually strive for improving and minimizing variability in postoperative outcomes. As the evidence base for the impact of social factors and patient and family engagement on health outcomes continues to evolve, further research should investigate seasonal and monthly variability in these factors.

## Conflicts of interest

The authors declare that there are no conflicts of interest.

For full disclosure statements refer to https://doi.org/10.1016/j.artd.2021.12.006.

## Supplementary data

Conflict of Interest Statement for Zalikha

Conflict of Interest Statement for Hussein

Conflict of Interest Statement for Abbas

Conflict of Interest Statement for El-Othmani

Conflict of Interest Statement for Karabon

## References

[1] HCUP Fast Stats (2021). http://www.hcup-us.ahrq.gov/faststats/national/inpatientcommonprocedures.jsp.

[2] Peel T.N., Cheng A.C., Liew D. (2015). Direct hospital cost determinants following hip and knee arthroplasty. Arthritis Care Res (Hoboken).

[3] Englesbe M.J., Pelletier S.J., Magee J.C. (2007). Seasonal variation in surgical outcomes as measured by the American College of Surgeons-National Surgical Quality Improvement Program (ACS-NSQIP). Ann Surg.

[4] Malik A.T., Azmat S.K., Ali A., Mufarrih S.H., Noordin S. (2018). Seasonal influence on postoperative complications after total knee arthroplasty. Knee Surg Relat Res.

[5] Ng M., Song S., George J. (2017). Associations between seasonal variation and post-operative complications after total hip arthroplasty. Ann Transl Med.

[6] Rosas S., Ong A.C., Buller L.T. (2017). Season of the year influences infection rates following total hip arthroplasty. World J Orthop.

[7] Bohl D.D., Fu M.C., Golinvaux N.S., Basques B.A., Gruskay J.A., Grauer J.N. (2014). The “July effect” in primary total hip and knee arthroplasty: analysis of 21,434 cases from the ACS-NSQIP database. J Arthroplasty.

[8] Rockov Z.A., Etzioni D.A., Schwartz A.J. (2020). The July effect for total joint arthroplasty procedures. Orthopedics.

[9] Tobert D.G., Menendez M.E., Ring D.C., Chen N.C. (2018). The “July effect” on shoulder arthroplasty: are complication rates higher at the beginning of the academic year?. Arch Bone Jt Surg.

[10] Lapointe-Shaw L., Austin P.C., Ivers N.M., Luo J., Redelmeier D.A., Bell C.M. (2018). Death and readmissions after hospital discharge during the December holiday period: cohort study. BMJ.

[11] Lenti M.V., Klersy C., Brera A.S. (2020). Clinical complexity and hospital admissions in the December holiday period. PLoS One.

[12] Phillips D.P., Jarvinen J.R., Abramson I.S., Phillips R.R. (2004). Cardiac mortality is higher around Christmas and New Year’s than at any other time: the holidays as a risk factor for death. Circulation.

[13] Houchens R., Elixhauser A. (2015). http://www.hcup-us.ahrq.gov/reports/methods/methods.jsp.

[14] Houchens R., Ross D., Elixhauser A. Final report on calculating National Inpatient Sample (NIS) variances for data years 2012 and later. 2015. HCUP Methods Series Report # 2015-09 ONLINE. December 14, 2015. U.S. Agency for Healthcare Research and Quality. http://www.hcup-us.ahrq.gov/reports/methods/methods.jsp.

[15] HCUP NIS Trend Weights (2015). http://www.hcup-us.ahrq.gov/db/nation/nis/trendwghts.jsp.

[16] Goodridge D., Henry C., Watson E. (2018). Structured approaches to promote patient and family engagement in treatment in acute care hospital settings: protocol for a systematic scoping review. Syst Rev.

[17] Cardoso-Moreno M.J., Tomas-Aragones L. (2017). The influence of perceived family support on post surgery recovery. Psychol Health Med.

[18] Wylde V., Kunutsor S.K., Lenguerrand E., Jackson J., Blom A.W., Beswick A.D. (2019). Association of social support with patient-reported outcomes after joint replacement: a systematic review and meta-analysis. Lancet Rheumatol.

[19] Lushniak B.D. (2013). Surgeon general’s perspectives. Public Health Rep.

[20] Bozic K.J., Ward L., Vail T.P., Maze M. (2014). Bundled payments in total joint arthroplasty: targeting opportunities for quality improvement and cost reduction. Clin Orthop Relat Res.

[21] Tarity T.D., Swall M.M. (2017). Current trends in discharge disposition and post-discharge care after total joint arthroplasty. Curr Rev Musculoskelet Med.

[22] Young J.Q., Ranji S.R., Wachter R.M., Lee C.M., Niehaus B., Auerbach A.D. (2011). “July effect”: impact of the academic year-end changeover on patient outcomes: a systematic review. Ann Intern Med.

[23] Crawford D.A., Berend K.R., Lombardi A.V. (2020). Fellow involvement in primary total knee arthroplasty: is there an “August effect?”. J Knee Surg.

[24] Dattilo J.R., Parks N.L., Ho H., Hopper R.H., McAsey C.J., Hamilton W.G. (2020). Does a “July effect” exist for fellowship training in total hip and knee arthroplasty?. J Arthroplasty.

[25] Pass H.I. (2010). Medical registries: continued attempts for robust quality data. J Thorac Oncol.

[26] Bozic K.J., Bashyal R.K., Anthony S.G., Chiu V., Shulman B., Rubash H.E. (2013). Is administratively coded comorbidity and complication data in total joint arthroplasty valid?. Clin Orthop Relat Res.

[27] Akinyemiju T., Meng Q., Vin-Raviv N. (2016). Association between body mass index and in-hospital outcomes: analysis of the nationwide inpatient database. Medicine (Baltimore).

[28] Browne J.A., Sandberg B.F., D’Apuzzo M.R., Novicoff W.M. (2014). Depression is associated with early postoperative outcomes following total joint arthroplasty: a nationwide database study. J Arthroplasty.

